# Expression of chitosanase from Aspergillus fumigatus chitosanase in Saccharomyces cerevisiae by CRISPR-Cas9 tools

**DOI:** 10.1186/s40643-023-00718-4

**Published:** 2024-02-02

**Authors:** Qingshuai Zhang, Hui Cao

**Affiliations:** Beijing Key Lab of Bioprocess, College of Life Science and Technology, Beijing University of Chemical Technology, Beijing, 100029 China

**Keywords:** Chitosan, Chitosanase, Saccharomyces cerevisiae, GTR-CRISPR

## Abstract

Chitooligosaccharides (COS) find numerous applications due to their exceptional properties. Enzymatic hydrolysis of chitosan by chitosanase is considered an advantageous route for COS production. Heterologous expression of chitosanase holds significant promise, yet studies using commonly employed *Escherichia coli* and *Pichia pastoris* strains encounter challenges in subsequent handling and industrial scalability. In this investigation, we opted for using the safe yeast strain Saccharomyces cerevisiae (GRAS), obviating the need for methanol induction, resulting in successful expression. Ultimately, utilizing the GTR-CRISPR editing system, shake flask enzyme activity reached 2 U/ml. The optimal chitosanase activity was achieved at 55℃ and pH 5, with favorable stability between 30 and 50 °C. Following a 2-h catalytic reaction, the product primarily consisted of chitobiose to chitotetraose, predominantly at the chitotriose position, with a slight increase in chitobiose content observed during the later stages of enzymatic hydrolysis. The results affirm the feasibility of heterologous chitosanase expression through Saccharomyces cerevisiae, underscoring its significant industrial potential.

**Graphical Abstract:**

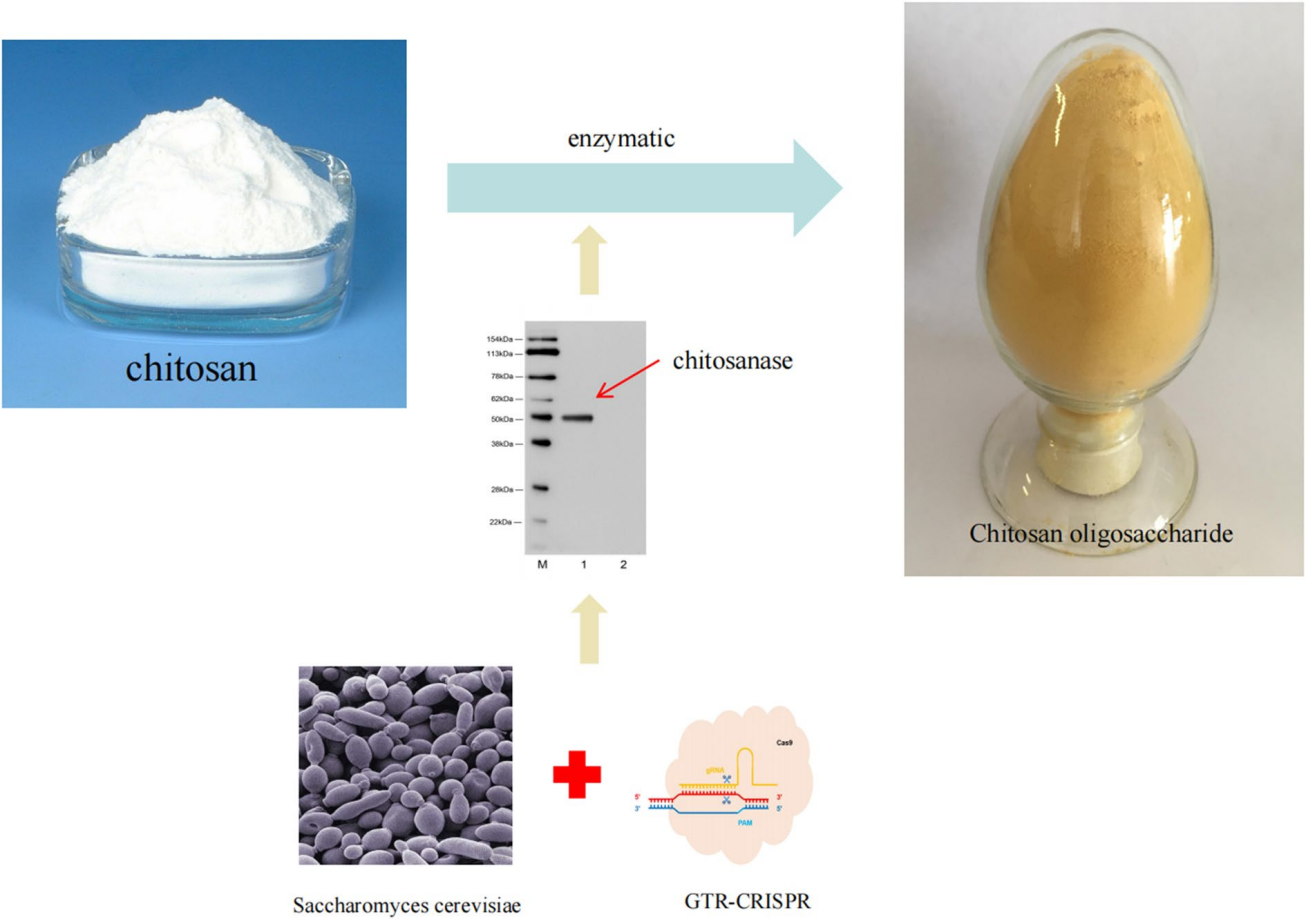

## Introduction

Chitosan emerges as a non-toxic and biocompatible cationic polysaccharide arising from the partial deacetylation of chitin. Chitosanase (EC 3.2.1.132) manifests the remarkable capability to catalyze chitosan, yielding the reduced polymerization degree of chito-oligosaccharides (COS). Chito-oligosaccharides exhibit distinctive attributes, including low molecular weight, high solubility in water, and notable biological activity. Presently, they have showcased substantial potential across a spectrum of applications, encompassing their potential as anticancer agents (Shen et al. 2009), their role as antimicrobial agents (Rhoades et al. 2006), their application in wound dressings(You et al. 2004), and their contribution to blood glucose reduction (Lee et al. 2003), among other functionalities.

Chitosanases (CSN) are primarily present in bacteria (Zhang and Sun 2007), fungi (Shimosaka et al. 1993), and a limited number of plants (Jung et al. 2006). The Carbohydrate-Active Enzymes (CAZy) database categorizes chitosanases into five distinct glycoside hydrolase families (GH): GH5, GH8, GH46, GH75, and GH80. Among these families, GH5 and GH8 exhibit chitosan hydrolytic activity and demonstrate the capability to degrade other glycosides, whereas GH46, GH75, and GH80 are dedicated chitosan-specific hydrolases (Ando et al. 2008; Pascal et al. 2015). These enzymes hold remarkable potential for further extensive inquiry and exploration.

The burgeoning potential application of chitooligosaccharides has sparked considerable interest in exploring chitosanases. The scientific literature has diligently pursued the expression of chitosanases across various host systems to achieve more optimal enzyme configurations. For instance, endeavors to express chitosanases within Escherichia coli have been undertaken; however, the ensuing CSN products manifest in the form of inclusion bodies, thus engendering intricate purification processes (Cheng et al. 2006). Alternatively, when the *Pichia pastoris* serves as the platform for CSN expression, the adoption of methanol, a potentially hazardous compound fraught with safety considerations, is often requisite as a carbon source and inducer during the fermentation process (Kang et al. 2012). When chitosan is used in food additives, agriculture, etc., downstream companies prefer performing it in a safer microbiological as well as fermentation environment.

In this study, we selected the yeast strain *Saccharomyces cerevisiae* as the host organism for expressing the endogenous chitosanase from *Aspergillus fumigatus* (GenBank Accession number AY190324), which has been identified for its potential in large-scale production of chitosan oligosaccharides (Cheng and Li 2000). The selected strain of *Saccharomyces cerevisiae* is classified as a GRAS (Generally Recognized as Safe) strain. The protein products can be secreted to the extracellular by adding signal peptides, which saves the cost of post-fermentation processing and does not require the addition of IPTG, methanol and other toxicity-inducing agents for cultivation during fermentation, which is conducive to the application of the product in the fields of food, medicine, agriculture, and enlarges its application. To enhance the efficiency and accuracy of subsequent experiments, we employed the gRNA-tRNA array for the CRISPR-Cas9 genome editing tool (GTR-CRISPR) to modify the genomic content of Saccharomyces cerevisiae (Yueping et al. 2019). The tool leverages the golden gate reaction to streamline the operational procedure and can concurrently edit genes at a minimum of two sites, thereby enhancing efficiency.

## Materials and methods

### Strains, plasmids and media

The yeast strain CEN.PK 113-5D *URA3Δ* employed in this study and the pCas plasmid were both maintained in our laboratory. Chitosan (degree of deacetylation ≥ 90%) and GlcN1-5 (chitooligosaccharide standards) were sourced from Qingdao BZ Oligo Biotech Co., Ltd. (China). Yeast strains were screened and cultured using SC-URA medium and YPD medium [1.0% (w/v) yeast extract, 2.0% (w/v) peptone, 2.0% (w/v) glucose].

### gRNA sequence

The gRNA sequences for the target sites used were predicted using an online tool (https://atum.bio/eCommerce/cas9/input). The gRNA sequences are listed in the Table 1.

**Table 1:** Sequences of gRNAs used in this study

Site	gRNA
X-2	ctctcgaagtggtcacgtgc
XII-2	tgaaactctaatcctactat

### Plasmid construction

The construction method for the pCas plasmid was adopted from the work of Professor Zhang Yueping (Yueping et al. 2019). The pCas plasmid features the SNR52 promoter as its flank, with BsaI cleavage sites located at the end of the SNR52 promoter (GATC) and the starting point of the gRNA scaffold (GTTT) on the SNR52 promoter. To assemble multiple gRNAs on a single plasmid, the lacZα sequence can be excised using BsaI and replaced with fragments obtained through PCR amplification.

### Preparation of dsDNA

The PCR reaction was carried out by mixing 10 μL Q5 enzyme reaction buffer, 10 μL of each of the two primers, 10 μL dNTP, 1 μL Q5 enzyme, and 9 μLddH2O. The PCR reaction conditions were set at 98 °C for 10 s, 58 °C for 20 s and 72 °C for 20 s, for a total of 35 cycles. PCR products were purified by ethanol precipitation and diluted with ddH2O at a concentration of 10 μg/μL. The synthesized donor was transformed into Saccharomyces cerevisiae by electrotransformation together with the tool plasmid.

### Preparation of crude enzymes and validation

The supernatant was concentrated by ultrafiltration through an ultrafiltration membrane (Pall Minimate TFF Capsule,10 k) to obtain a solution with a high concentration of the target protein, followed by western blotting to detect the expression of the target protein with the strep tag II(Zhiqiang et al. 2008).

### Measurement of enzyme activity

Chitosanase activity was measured by the dinitrosalicylic acid method. A 1% chitosan solution was initially prepared using a sodium acetate buffer (50 mM, pH 5). This solution was then combined in a 1:1 ratio with samples prepared using the same buffer solution (final volume 2 ml) and subjected to a 15-min reaction at the optimum temperature. Afterward, the reaction was terminated by adding 2 ml of DNS (3,5-dinitrosalicylic acid). The mixture was then placed in a water bath at 100 °C for 10 min, after which it was cooled to room temperature and the absorbance was measured at 540 nm. One unit (U) of chitosanase is defined as the amount of chitosanase that releases 1 μmol of reducing sugar per minute. Glucosamine was used as a standard. The experiments were done in triplicate and the average values are reported (Cheng and Li 2000).

### Effects of pH and temperature on the recombinant chitosanase activity and stability

To investigate the impact of pH on enzyme stability, various buffer solutions were employed for enzyme incubation: Glycine/HCl 50 mM, pH 2.0–3.5; Acetic acid/NaAc 50 mM, pH 3.5–6.0; Phosphate buffer 50 mM, pH 6.0–7.0; Tris–HCl 50 mM, pH 7.0–9.0; Gly-NaOH 50 mM, pH 9–10. The enzyme was then mixed at a ratio of 1:49 with these buffers and incubated at 25 °C for 1 h. Subsequently, residual enzyme activity was assessed to ascertain pH stability, and the enzyme activity at pH = 5 was set to 100%. To ascertain the optimal pH for *chitosanase* (CSN), a 1% chitosan solution was prepared in the aforementioned buffer solution. Subsequently, enzyme activity was measured at 55 °C across a pH range of 3.0 to 8.0 over a duration of 15 min. The highest enzyme activity detected was set to 100%.

To determine the stability of CSN in different temperature ranges (30 °C–80°C), residual enzyme activity was measured by incubation at different temperatures for 1 h. After 1 h of incubation at 30 °C, the enzyme activity was set to 100%. The optimal temperature for the enzyme was determined simultaneously in the temperature range of 30–80 °C, with the highest detected enzyme activity set at 100%.

### Effects of different metal ions on the activity of recombinant chitosanase

To investigate the impact of metal ions on the activity of recombinant chitosanase, the assay was conducted following the aforementioned procedure for a duration of 1 h. Various metal ions, including Zn2 + , Cu2 + , Mg2 + , Fe3 + , Ca2 + , K + , and Mn2 + , were introduced to attain a final concentration of 10.0 mM.

### Analysis of enzyme digestion products

To analyze the enzymatic products, 1 ml of a mixture containing 1% chitosan and chitosanase was incubated at 60 °C. The reaction products were taken at different times and analyzed by thin-layer chromatography (TLC, thin-layer chromatography) on silica gel plates. The spreading agent was n-propanol, 25% (v/v) ammonia (2:1). A 0.1% ethanolic solution of ninhydrin was used as the chromogenic agent, and the color development conditions were 105 °C for 5 min.

## Results and discussion

### Strain construction and screening

**Fig.1:**
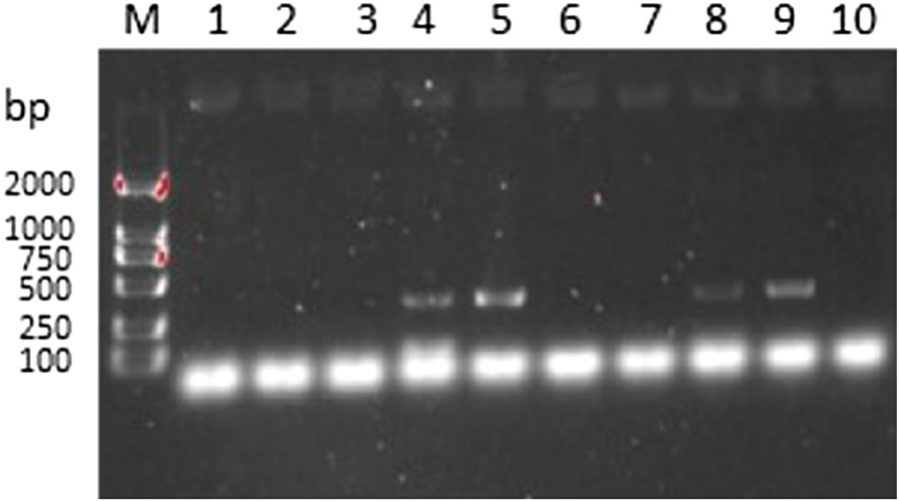
Analysis of DNA gel electrophoresis for colony PCR fragments. Lane M, DNA marker; Lanes 1–5: Validation of X-2 site; Lanes 6–10: Validation of insertion at XII-2 site; Bands appear in both Lane 4 and Lane 9, indicating that the strain in question is a biallelic yeast strain

The pCas plasmid used was constructed by Golden Gate assembly, and was transferred into Saccharomyces cerevisiae (CEN.PK 113-5D ΔURA3) together with the treated chitosanase gene (GenBank: AY190324) fragment of *Aspergillus fumigatus* origin, and screened by incubation on SC-URA nutrient-deficient medium, and finally, by PCR on five colonies screening (Fig. 1) to obtain two-copy yeast strains. In the construct, we also obtained single-copy organisms (Fig. 1, colonies corresponding to lanes 5 and 10), whose crude enzyme activity (0.15 U/ml) was detected to be much lower compared to the two-copy, and we subsequently used the two-copy strains for subsequent experiments.

When reviewing the literature, we found that some researchers transformed plasmids with different copy numbers into the defatted yeast, and the enzyme expression level of the enzyme activity with a higher copy number had a particular advantage relative to that of the low-copy one, but the higher copy number may also produce a higher pressure on the Saccharomyces cerevisiae cells, which in turn reduces the expression level, and it is necessary to do further optimization of these in the future research. These need to be further optimized in future studies to increase the expression level further.

### Expression and characterization of chitosanase

**Fig. 2:**
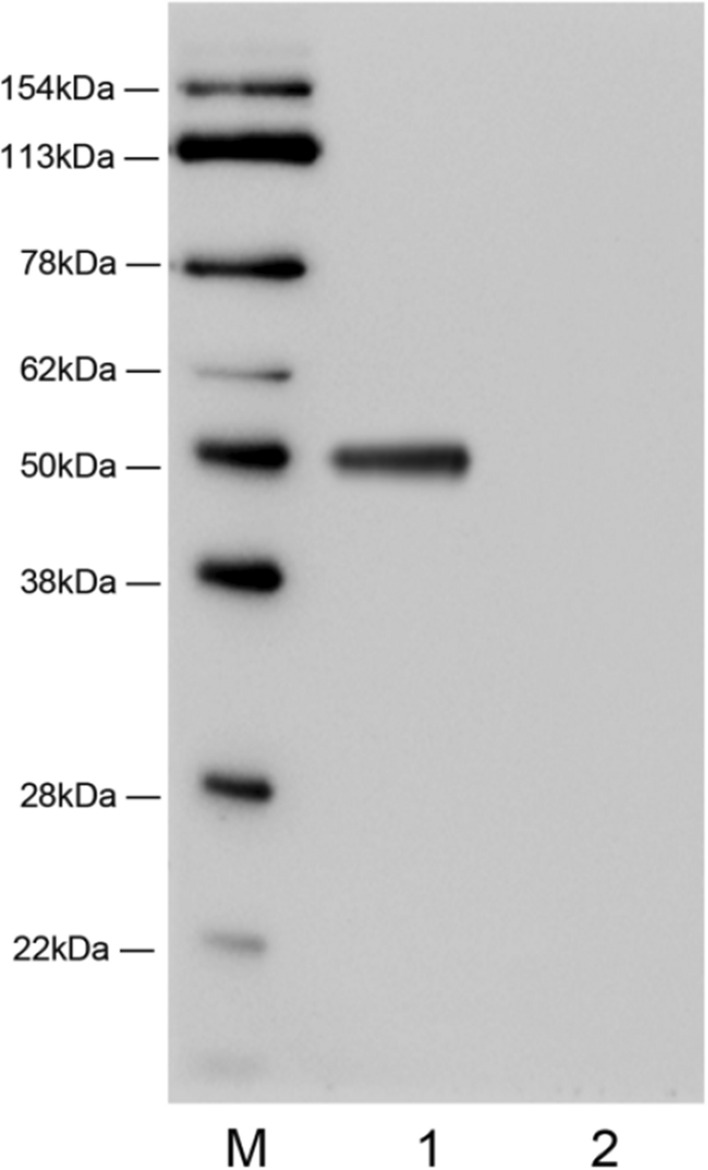
Western blot analysis of recombinant chitosanase. Lane M, molecular weight protein markers; Lane 1, fermentation supernatant; Lane 2, fermentation broth precipitate

Following the removal of the pCas plasmid, the recombinant yeast strain underwent cultivation in the YPD medium. Subsequently, the resultant supernatant underwent concentration via ultrafiltration using a 10 k Pall Minimate TFF Capsule. The enzyme activity within the clarified supernatant was determined to be 2 U/ml. A Strep-Tag II sequence was integrated into the protein structure, facilitating the validation of target protein expression through protein blotting analysis. The protein blotting findings (depicted in Fig. 2) unveiled distinct bands approximately at 50 kDa within the supernatant, thereby affirming the successful manifestation of the intended protein.

A comparison of chitosanase activities from several different sources shows (Table 2) that the chitosanase gene in this study has a higher enzyme activity (2 U/ml) in Saccharomyces cerevisiae than other sources of chitosanase, suggesting that the use of Saccharomyces cerevisiae as a host strain holds potential.

**Table 2:** Chitosyase activity produced by different strains

Strain	The chitosanase activity	References
*Amycolatopsis* sp. CsO-2	1.6U/ml	(Okajima et al. 1994)
*Serratia marcescens* TKU011	0.5U/ml	(Wang et al. 2008)
*Bacillus* sp. KCTC 0377BP	1.2U/ml	(Choi et al. 2004)
*Bacillus* sp. TKU007	0.4U/ml	(San-Lang et al., 2008)

### Effects of different temperatures and pHs on activity and stability of the recombinant chitosanase

**Fig. 3:**
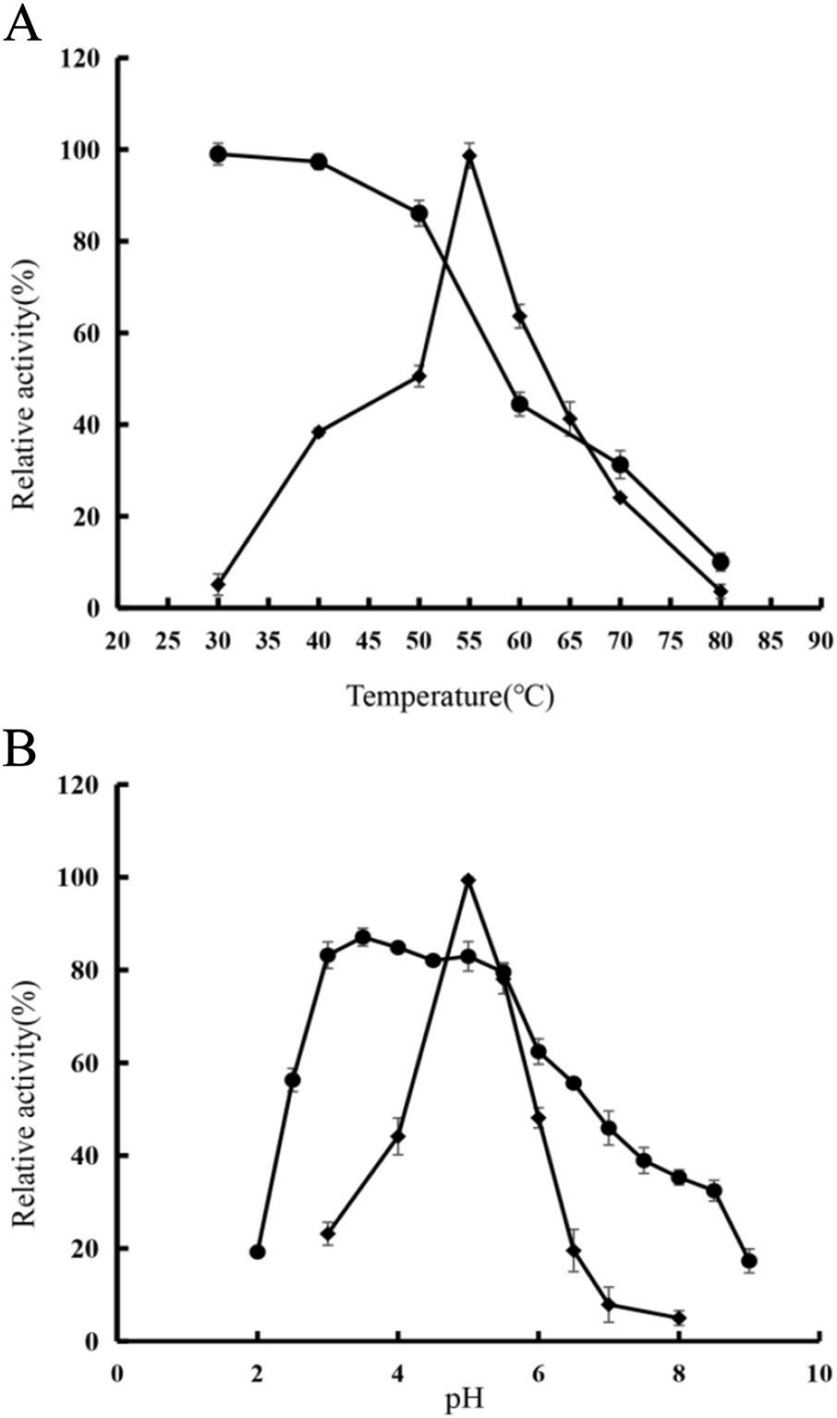
Effect of different temperatures (**A**) and pH (**B**) on CSN activity (solid diamonds) and stability (solid circles). Inactivated samples were used as controls. Data are given as mean ± standard deviation, *n* = 3

As shown in Fig. 3A, chitosanase has an optimal temperature at 55 °C. The thermal stability of the enzyme was analyzed for incubation in the range of 30–80 °C for 1 h. The results show that the enzyme maintains a good stability up to 50 °C. Notably, the temperature increase to 60 °C protein also retains about 40% of the activity and keeps more than 80% of the activity below 50 °C. Higher reaction temperature and stability can accelerate the reaction, reduce the viscosity of the substrate solution, and inhibit the growth of microorganisms to a certain extent (Zamost et al. 1991), which has great potential for industrial applications.

The optimal pH for recombinant chitosanase was 5 (Fig. 3B). Recombinant chitosanase remained active for more than 60% for 1 h in the pH range of 3–6 (Fig. 3B). The loss of enzyme activity was more pronounced when the pH exceeded 5.5. The optimal pH for chitosanase is generally in the acidic range (pH 4–7) (Ando et al. 2008; Guo et al. 2019), probably due to chitosan’s acid-soluble and base-insoluble nature.

Recombinant chitosanases acquired through this methodology exhibit notable advantages in terms of thermal stability when juxtaposed with the majority of heterologously expressed chitosanases. For instance, the chitosanase derived from E. coli by Johnsen et al. demonstrated a relative enzyme activity below 20% at 70 °C, a value notably lower than the 30% depicted in Fig. 3A (Johnsen et al. 2010). However, it must be noted that the thermal stability of the recombinant chitosanase was slightly reduced compared to that of the native chitosanase of *Aspergillus fumigatus* Y2K. The original chitosanase from *A. fumigatus* Y2K maintained a relative enzyme activity exceeding 45% within the temperature range of 65–70 °C (Cheng and Li 2000). This alteration could be linked to the molecular weight of the protein illustrated in Fig. 2 (approximately 50 kDa), surpassing the theoretical molecular weight of 25 kDa. The analysis may be the effect of the glycosylation of brewer's yeast and the future needs to be further research on the role of modification of proteins in the brewer's yeast to address the effect of glycosylation on the properties of chitosanase to exploit its potential better. Thus, its potential can be better utilized.

### Effect of different cations on the activity of chitosanase

To study the effect of metal ions on enzyme activity at certain concentrations, the residual enzyme activity was assayed by incubation at a final concentration of metal ions of 10 mM pH = 5 (acetic acid/sodium acetate) at a temperature of 30 °C for 1 h. As shown in Table 3, Cu^2+^, Fe^3+^ and Zn^2+^ at a final concentration of 10 mM had high inhibitory activities on the recombinant enzyme. Interestingly, the enzyme activity was significantly increased by adding 10 mM Mn^2+^. The enzyme activity measured without the ions used in the experiment was taken as 100%.

**Table 3:** presents the impact of various metal ions on chitosanase activity

Cation	Relative activity (%)
Mn^2+^	125 ± 1.2
Mg^2+^	80 ± 0.5
Cu^2+^	60 ± 0.7
Fe^3+^	55 ± 0.8
Zn^2+^	65 ± 0.6
Ca^2+^	71.5 ± 0.5
K^+^	82.25 ± 0.8

Data are presented as mean ± standard deviation, with n = 3

In the study of the effect of metal ions on chitosanase, it was found that Mn^2+^ ions could enhance the activity of chitosanase. However, other metal ions (e.g., Cu^2+^, Fe^3+^, and Zn^2+^, etc.) had an inhibitory effect on its activity. The analysis may be that chitosanase has metal ion binding sites and Mn^2+^ may bind to specific sites, contributing to increased spatial stability and catalytic activity (Yue et al. 2019). The effect of Mn^2+^ on chitosanase from different sources varies. For example, chitosanase produced by Salmonella is completely inhibited by Mn^2+^ (Wang et al. 2008), as well as recombinant chitosanase from Pseudomonas aeruginosa sources are also inhibited by inhibition by Mn^2+^ (1.0 mM) (Ando et al. 2008). The difference in response to the same metal ions may be due to some specific spatial structure differences, and future structural studies may be better able to help in its better application in production.

### Analysis of enzymatic products

**Fig. 4:**
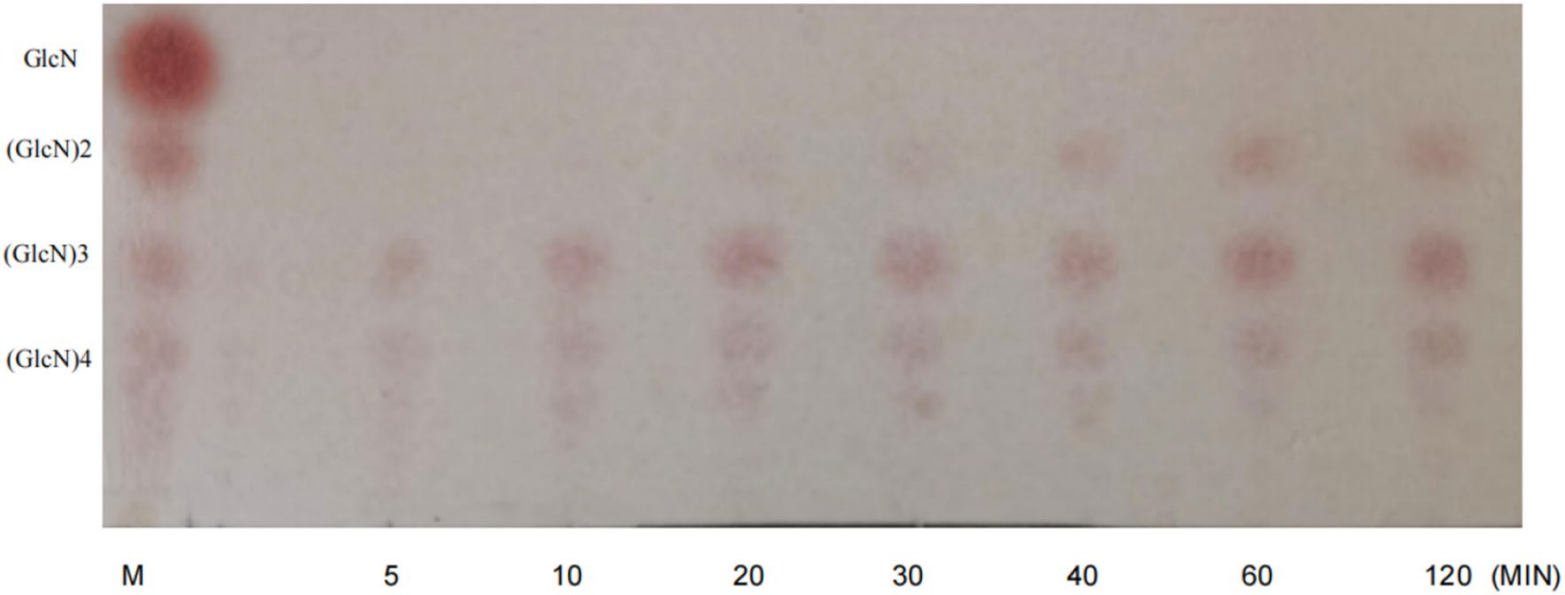
TLC analysis of hydrolysis products over time. M: Chitooligosaccharide mixed standard

The reaction was performed at 55 °C using 1% (w/v) chitosan solution as substrate and samples were taken periodically for analysis. The results of thin layer chromatography (TLC) showed (Fig. 4) that the hydrolysis products consisted of (GlcN)2 to (GlcN)4. We roughly calculated the percentage of oligosaccharides in the grey values in Fig. 4 by software, and (GlcN)3 was about 45% at 120 min, and (GlcN)2 content was about 15% based on the grey values.

Chitosanases are classified as endonucleases and exonucleases depending on the mode of digestion, and the final product of the chitosanase used in this study consisted of (GlcN)2 to (GlcN)4, which indicates that it is an endonucleating chitosanase. In the enzyme digestion process, the products mainly consisted of (GlcN)3 to (GlcN)4, while (GlcN)2 existed in a smaller proportion and appeared later in the early stage. The original strain of this chitosanase also had a similar situation, and it was analyzed that the special spatial structure of this chitosanase might have led to the special result of the enzyme products (Cheng and Li 2000). It was analyzed that the special spatial structure of the chitosanase might have led to such a special result (Han et al., 2014).

## Conclusions

In summary, we finally expressed the chitosanase gene of *Aspergillus fumigatus* origin in Saccharomyces cerevisiae by CRISPR/Cas9 tool, and the recombinant protein obtained maintained the optimal enzyme activity of 2U/ml at pH = 5 temperature 55 °C and high stability at temperature below 50 °C. It was found that Mn2 + could improve the enzyme activity to a certain extent in the metal ion experiment, which provided a new idea for its application. The study of its enzymatic products found that they were mainly composed of (GlcN)2 to (GlcN)4, which had typical endonuclease characteristics. In conclusion, despite the impact of *Saccharomyces cerevisiae* on protein over-modification, the expression of chitosanase in this yeast demonstrates notable safety features. Additionally, the fermentation process in *Saccharomyces cerevisiae* is characterized by the absence of harmful substance production. Consequently, chitosanase expression in *Saccharomyces cerevisiae* holds significant promise for widespread utilization in diverse fields such as agriculture, food, and medicine, offering extensive prospects for applications.

## Data Availability

All data generated or analyzed during this study are included in this article.

## Abbreviations

COS: Chitooligosaccharides
GRAS: Generally recognized as safe
GTR-CRISPR: A gRNA-tRNA array for CRISPR-Cas9
TLC: Thin-layer chromatography
